# Health-related quality of life in patients with unresectable hepatocellular carcinoma treated with SIRT and nivolumab: a sub-analysis of the NASIR-HCC trial

**DOI:** 10.1186/s41687-025-00873-6

**Published:** 2025-04-08

**Authors:** Manuel De la Torre-Aláez, Ana Matilla, María Varela, Mercedes Iñarrairaegui, María Reig, José Luis Lledó, Juan Ignacio Arenas, Sara Lorente, Milagros Testillano, Laura Márquez, Gemma Iserte, Josepmaria Argemí, Carlos Gómez-Martin, Macarena Rodríguez-Fraile, José I. Bilbao, Richard F. Pollock, Johannes Pöhlmann, Ion Agirrezabal, Bruno Sangro

**Affiliations:** 1 Liver Unit and HPB Oncology Area, Clínica Universidad de Navarra, Madrid, Spain; 2 Centro de Investigación Biomédica en Red de Efermedades Hepáticas y Digestivas (CIBEREHD), Madrid, Spain; 3 Digestive Diseases Service, Hospital General Universitario Gregorio Marañón, Madrid, Spain; 4 Liver Unit, Hospital Universitario Central de Asturias, IUOPA, ISPA, Universidad de Oviedo, Oviedo, FINBA Spain; 5 Liver Unit and HPB Oncology Area, Clinica Universidad de Navarra, Pamplona, Spain; 6 Liver Oncology Unit, Liver Unit, ICMDM, Hospital Clinic, Barcelona, Spain; 7 BCLC Group, IDIBAPS, Universidad de Barcelona, Barcelona, Spain; 8 Gastroenterology and Hepatology Service, Hospital Universitario Ramon y Cajal, IRYCIS, Universidad de Alcalá, Madrid, Spain; 9 Digestive Diseases, Hospital Universitario Donostia, San Sebastián, Spain; 10 Liver Unit, Hospital Clínico Lozano Blesa, Zaragoza, Spain; 11 Digestive Diseases, Hospital Universitario de Cruces, Baracaldo, Spain; 12 Medical Oncology, Hospital Universitario, 12 de Octubre, Madrid, Spain; 13 Nuclear Medicine, Clínica Universidad de Navarra, Pamplona, Spain; 14 Interventional Radiology, Clínica Universidad de Navarra, Pamplona, Spain; 15 Covalence Research Ltd, Rivers Lodge, West Common, Harpenden, AL5 2JD UK; 16 Sirtex Medical Europe GmbH, Bonn, Germany

**Keywords:** Hepatocellular carcinoma, Quality of life, Selective internal radiation therapy, SIR-Spheres, Nivolumab, Immunotherapy

## Abstract

**Background:**

The health-related quality of life (HRQoL) impact of therapies for hepatocellular carcinoma (HCC) influences decision-making and treatment outcomes. The present study reports HRQoL results from NASIR-HCC, a single-arm study of selective internal radiation therapy (SIRT) with Y90 resin microspheres followed by nivolumab for unresectable HCC.

**Methodology:**

Participants completed the EQ-5D-3 L, EQ-VAS, and FACT-Hep at baseline and on the first day of each nivolumab cycle. Linear mixed-effect models were used to calculate changes in outcomes in participants with the baseline and ≥ 1 follow-up measurement. Changes were assessed for clinical meaningfulness versus published minimally important differences.

**Results:**

Thirty-two patients from NASIR-HCC were included. Completion rates exceeded 70% at 62% of time points. Across EQ-5D-3 L domains, minimal changes were reported. Most patients had no problems at almost all time points. Mean index values were 0.864 at baseline and 0.763 in cycle 8, but this difference was not clinically meaningful. The small EQ-VAS increase, from 74.8 at baseline to 75.9 in cycle 8, was also not clinically meaningful. The various FACT scales remained stable, although transient but not clinically meaningful declines occurred for some scales. The median time to deterioration was 5.5 months for the FACT-Hep score.

**Conclusions:**

Combining SIRT with nivolumab did not compromise HRQoL in patients with unresectable HCC. Study results were limited by the small number of patients but, combined with the previously reported clinical outcomes, suggested that the treatment combination deserves further consideration in this difficult-to-treat population.

**Trial registration number/date of registration:**

NCT03380130. First submitted on 2017-10-20; https://clinicaltrials.gov/study/NCT03380130.

**Supplementary Information:**

The online version contains supplementary material available at 10.1186/s41687-025-00873-6.

## Abstract (plain language summary)

Patients with hepatocellular carcinoma often have poor health-related quality of life. Any treatment for hepatocellular carcinoma should aim to, at best, improve health-related quality of life, and, at the very least, prevent further declines. For the novel treatment combination of selective internal radiation therapy with Y90 resin microspheres and the immunotherapeutic drug nivolumab, clinical but no health-related quality of life data have been published before. The present study is the first to provide such data, based on the NASIR-HCC study, in which participants with unresectable hepatocellular carcinoma completed multiple instruments to measure health-related quality of life, including the EQ-5D-3 L and the FACT-Hep questionnaire. For all measurements, scores remained broadly stable over time and where changes were observed, in either a positive or a negative direction, these were transient and not clinically meaningful. These findings imply that selective internal radiation therapy followed by eight cycles of nivolumab does not reduce health-related quality of life in unresectable hepatocellular carcinoma. This treatment combination therefore should be evaluated further for this patient population, in particular as its clinical data have also been promising.

**Supplementary Information:**

The online version contains supplementary material available at 10.1186/s41687-025-00873-6.

## Background

Liver cancer is the third-ranked cause of cancer death globally and accounts for 8.3% of all cancer deaths [1]. Hepatocellular carcinoma (HCC) accounts for 80–90% of liver cancer cases and occurs frequently in patients with chronic liver disease [2]. The etiology of liver diseases varies by geographic area, from predominantly hepatitis B/C viral infections to excessive alcohol intake and metabolic dysfunction-associated steatotic liver disease. Most patients are diagnosed at intermediate or advanced stages, for which the main treatment options are intraarterial and systemic therapies [2, 3]. Transarterial chemoembolization (TACE) is the most common form of intraarterial therapy, but not recommended in the presence of a high tumor burden or portal vein invasion [4]. Alternatively, selective internal radiation therapy (SIRT), also referred to as radioembolization, can be considered. With SIRT, radioactive microspheres loaded with yttrium-90 (Y90) are injected into the hepatic arteries [5]. SIRT has no significant ischemic effect [6] so can be applied in patients who are not good candidates for TACE [7].

In the ongoing search for improvements to HCC treatment, immunotherapies in general and immune checkpoint inhibitors (ICIs) in particular have been identified as promising treatment options [8]. Recently, the combinations of atezolizumab with bevacizumab [9] or tremelimumab with durvalumab [10] have become the standard of care first-line treatment for HCC patients at Barcelona Clinic Liver Cancer (BCLC) stage C or BCLC stage B not amenable for TACE [11]. Other immunotherapies have also been investigated, including nivolumab, which showed durable objective response rates in a phase 1/2 trial in advanced HCC [12] and overall survival (OS) comparable to sorafenib, with a good safety profile, versus sorafenib in phase 3 trial [13]. Recently, the phase 3 trial CheckMate 9DW reached its primary endpoint of prolonged OS with nivolumab plus ipilimumab compared to lenvatinib or sorafenib [14]. As SIRT has immunogenic effects [15, 16], combinations of immunotherapies with SIRT have also been investigated, including SIRT with Y-90 resin microspheres (SIR-Spheres^®^) followed by nivolumab in the single-arm CA 209–678 and NASIR-HCC trials [17, 18]. In these studies, the sequence of SIRT and nivolumab had an acceptable safety profile and demonstrated antitumor activity in patients with unresectable, liver-only HCC who, in the NASIR-HCC trial, were not eligible for TACE and free from extrahepatic metastasis.

The NASIR-HCC study also collected information on health-related quality-of-life (HRQoL). Quality of life is increasingly acknowledged as an important outcome in clinical studies in HCC that reflects the significant symptom burden of the disease [19], but previous reviews have noted that corresponding data remain sparse, especially for advanced HCC [20, 21]. The present study aims to expand on the available HRQoL evidence by reporting the effects of SIRT plus nivolumab on symptom, HRQoL, and health status trajectories and by providing estimates of time to treatment deterioration (TTD), while describing the patient-reported burden of select symptoms based on the NASIR-HCC study. Based on the findings described above, notably regarding the good safety profile of SIRT plus nivolumab, it was anticipated that HRQoL would not be compromised but remain largely unchanged following treatment with SIRT plus nivolumab in unresectable HCC.

## Methods

NASIR-HCC was a single-arm, open-label, phase 2 trial in 42 patients with unresectable HCC free from distant metastasis who were candidates for loco-regional therapy but not good candidates for TACE, conducted as a multicenter study in Spain. Patients with BCLC B2 stage or predominantly unilobar tumors with segmental or lobar portal vein invasion were treated with SIRT using SIR-Spheres^®^ Y90 resin microspheres, followed 3 weeks later by 240 mg nivolumab given every 2 weeks until completion of eight cycles (each one consisting of three doses, for a total of twenty-four doses), disease progression, or unacceptable toxicity [18]. All SIRT procedures were done at the same center (Clínica Universidad de Navarra), as single-day procedures, to ensure consistent treatment. The study design as well as safety and effectiveness results of the NASIR-HCC study are described in detail by de la Torre-Aláez et al. [18].

### Patient-reported outcome instruments used in NASIR-HCC

The NASIR-HCC study also assessed participants’ HRQoL at baseline and over the treatment course. Participants were asked at baseline and on the first day of each nivolumab cycle to complete two patient-reported outcomes measures (PROMs). Firstly, the EQ-5D-3 L descriptive system and the EQ visual analogue scale (EQ VAS) were used. The EQ-5D-3 L descriptive system includes five dimensions (mobility, self-care, usual activities, pain/discomfort, anxiety/depression), each with three levels (no, some, or extreme problems). Based on digits associated with each response, a combined 5-digit number is created that reflects a participant’s health state. The EQ VAS allows the patient to self-rate their health on a scale ranging from “Best imaginable health state” to “Worst imaginable health state”.

Secondly, disease-specific symptoms and functioning were assessed using the Functional Assessment of Cancer Therapy-Hepatobiliary (FACT-Hep), a questionnaire combining a disease-specific, hepatobiliary cancer subscale (HCS; 18 items; scores ranging from 0 to 72 points) with the FACT-General (FACT-G; 27 items; scores ranging from 0 to 108 points) questionnaire [22]. FACT-G includes multiple HRQoL domains relevant to cancer, namely physical wellbeing (PWB; scored between 0 and 28 points), emotional wellbeing (EWB; 0–24 points), social and family wellbeing (SWB; 0–28 points), and functional wellbeing (FWB; 0–28 points). Each of these wellbeing scales contains several items rated on a 5-point Likert scale ranging from 0 (“not at all”) to 4 (“very much”). Throughout, higher scores indicate better health status.

### Statistical analyses

The HRQoL analyses were performed on all participants with a valid baseline assessment and ≥ 1 post-baseline assessment, for up to eight nivolumab cycles. The thresholds for clinically meaningful change were pre-specified based on published minimally important differences (MIDs) for each instrument, as ≥ 2 points for the EWB, FWB, PWB, SWB scales, ≥ 5 points for the HCS, ≥ 6 points for FACT-G, and ≥ 8 points for FACT-Hep [23]. For index values derived from the EQ-5D-3L, the MID was specified as ≥ 0.1, for the EQ VAS as ≥ 8 points [24].

Descriptive statistics were generated for the FACT-Hep and EQ-5D-3L at all assessment time points and plotted over time. Mixed-effects linear models were used to assess changes for each FACT-Hep subscale and for EQ-5D-3L measurements. Unadjusted models included HRQoL measurements as a dependent variable, with time (cycle number) as the explanatory variable and a random intercept for patients. Models were subsequently adjusted for age and Eastern Cooperative Oncology Group (ECOG) performance status (adjusted models are reported in detail in the supplementary appendix). Statistical significance was assessed versus a Bonferroni-corrected alpha value of 0.003. Model fit was evaluated with Akaike and Bayesian information criteria (AIC and BIC), and likelihood ratio tests. Time to deterioration (TTD) was evaluated for HCS and FACT-Hep total scores and defined as the time from baseline to the first decrease in HRQoL equal to or exceeding the corresponding MID.

Analyses were conducted in R version 4.0.3, including the *eq5d* package to convert EQ-5D responses into utility values [25], while R version 4.3.3 and the *ggplot2* package [26] were used for generating plots.

## Results

### Analysis sample and instrument completion rates

In the NASIR-HCC trial, the 42 participants receiving SIRT were defined as the safety population (one of whom did not receive nivolumab due to an adverse event related to SIRT) [18]. Ten participants in the safety population did not complete either HRQoL instrument at baseline, thereby limiting the present analysis to 32 evaluable participants with FACT-Hep and EQ-5D-3 L completed at baseline. The median age of these participants was 62 years, and most had BCLC stage B tumors (Table 1).

**Table 1:** Baseline characteristics, treatment duration, and time to progression

Characteristic, outcome	Values	
Participants, n (%)	32 (100)	
Males, n (%)	27 (84.4)	
Age, median (interquartile range)	65 (50 to 79)	
Barcelona Clinic Liver Cancer stage, n (%)	A	2 (6.4)
	B	18 (56.3)
	C	12 (37.3)
Etiology, n (%)	Uninfected	22 (68.7)
	Viral hepatitis C	10 (31.3)
Eastern Cooperative Oncology Group performance status, n (%)	0	29 (90.6)
	1	3 (9.4)
Child Pugh score, n (%)	A5	26 (81.25)
	A6	6 (18.75)
Albumin-bilirubin grade, n (%)	1	17 (53.13)
	2	15 (46.87)
Alpha fetoprotein > 400, n (%)	> 400	10 (31.25)
	*≤* 400	22 (68.75)
Selective internal radiation therapy target, n (%)	Sublobar	4 (12.5)
	Lobar	22 (68.75)
	Whole liver	6 (18.75)
Weeks on nivolumab, median (range)	28.7 (3.8 to 48.8)	
Discontinued nivolumab, n (%)	27 (84.3)	
Months to progression, median (95% confidence interval)	8.1 (5.6 to 10.5)	

In this sample, the rate of PROM completion was > 70% (the expected threshold for meaningful interpretation) in 62% of time points and was > 60% in 98% of time points. Analyses of TTD could be conducted for 15 events observed in 26 respondents evaluable for the FACT-Hep total score and 10 events in 26 respondents evaluable for the HCS.

### Patient-reported outcomes

#### EQ-5D

**Fig. 1:**
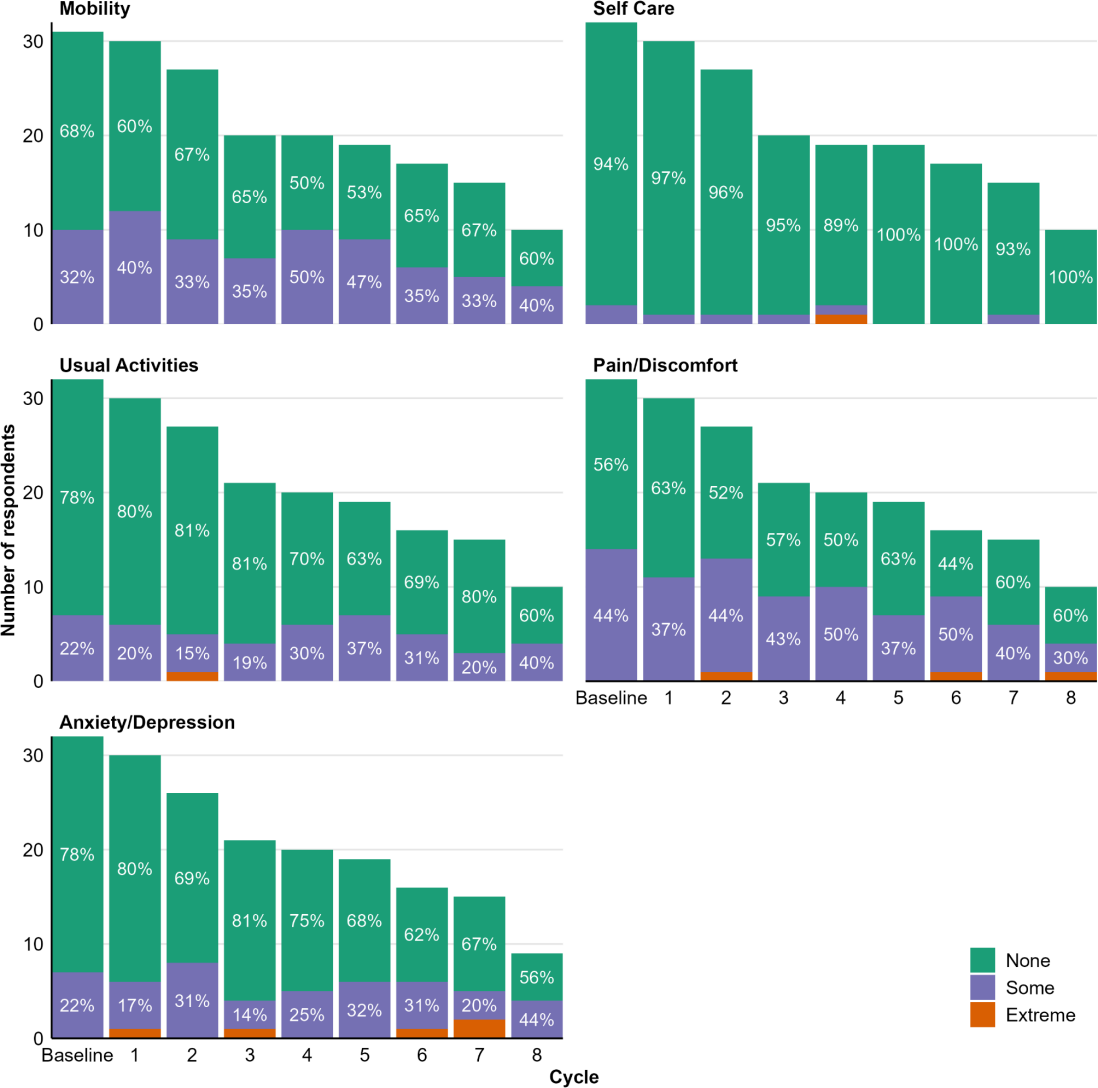
EQ-5D-3L cohort profile over treatment cycles. Values in bars indicate the percentage of respondents per cycle. Segments displaying values ≤ 10% are not labeled but can be inferred as the complement of labeled values. In cycle 4, 5% of respondents reported “some” and “extreme” problems with self-care, respectively

Domains in the EQ-5D-3L descriptive systems showed minimal change during the treatment period (Fig. 1). Most respondents reporting “extreme” problems did so for the anxiety/depression domain (ranging between 3.3% at visit 1 to 13.3% at visit 7), but the overall number of responses indicating “extreme” problems at any time point was small, with one patient each reporting extreme problems in usual activities at visit 2, in self-care at visit 4, and pain/discomfort at visits 2,6, and 8. In all domains and at nearly all time points, more than 50% of participants reported no problems, except for pain/discomfort for which 6% and 50%, respectively, reported “extreme” or “some” problems in cycle 6.

**Fig. 2:**
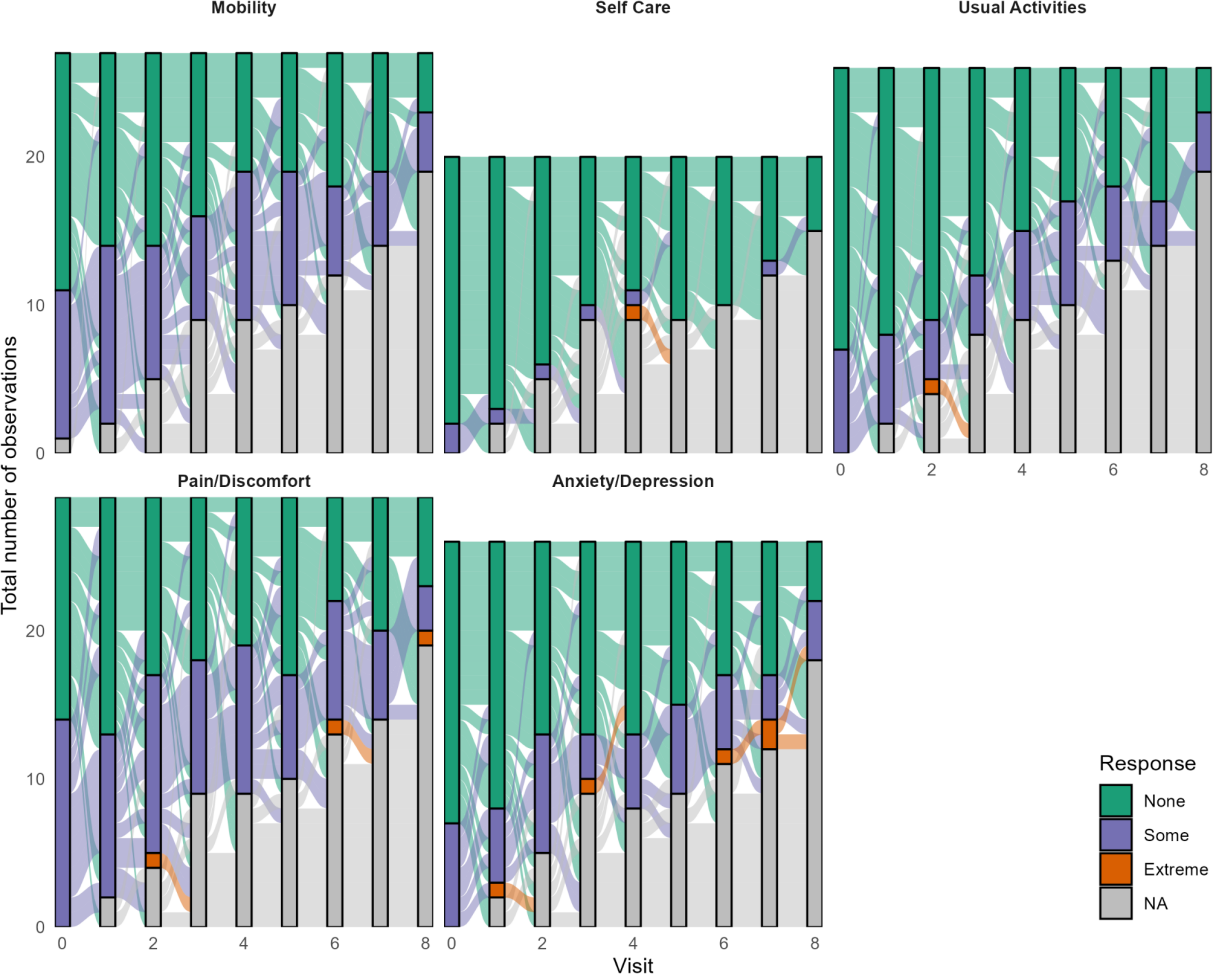
EQ-5D-3L response trajectories. Values on the vertical axis indicate the total number of observations. “NA” is “not available” due to non-completion of a domain by the participant for a given visit

Investigating individual EQ-5D-3L response profiles over time showed that participants tended to respond consistently over time, i.e., to report the same level of problems at subsequent visits as for the initial visit (Fig. 2). Participants reporting “extreme” problems at any time point tended to discontinue the study, so often had no subsequent response available. However, where a subsequent response was available (in the Anxiety/Depression domain), problems improved over time, including patients subsequently reporting no problems.

**Fig. 3:**
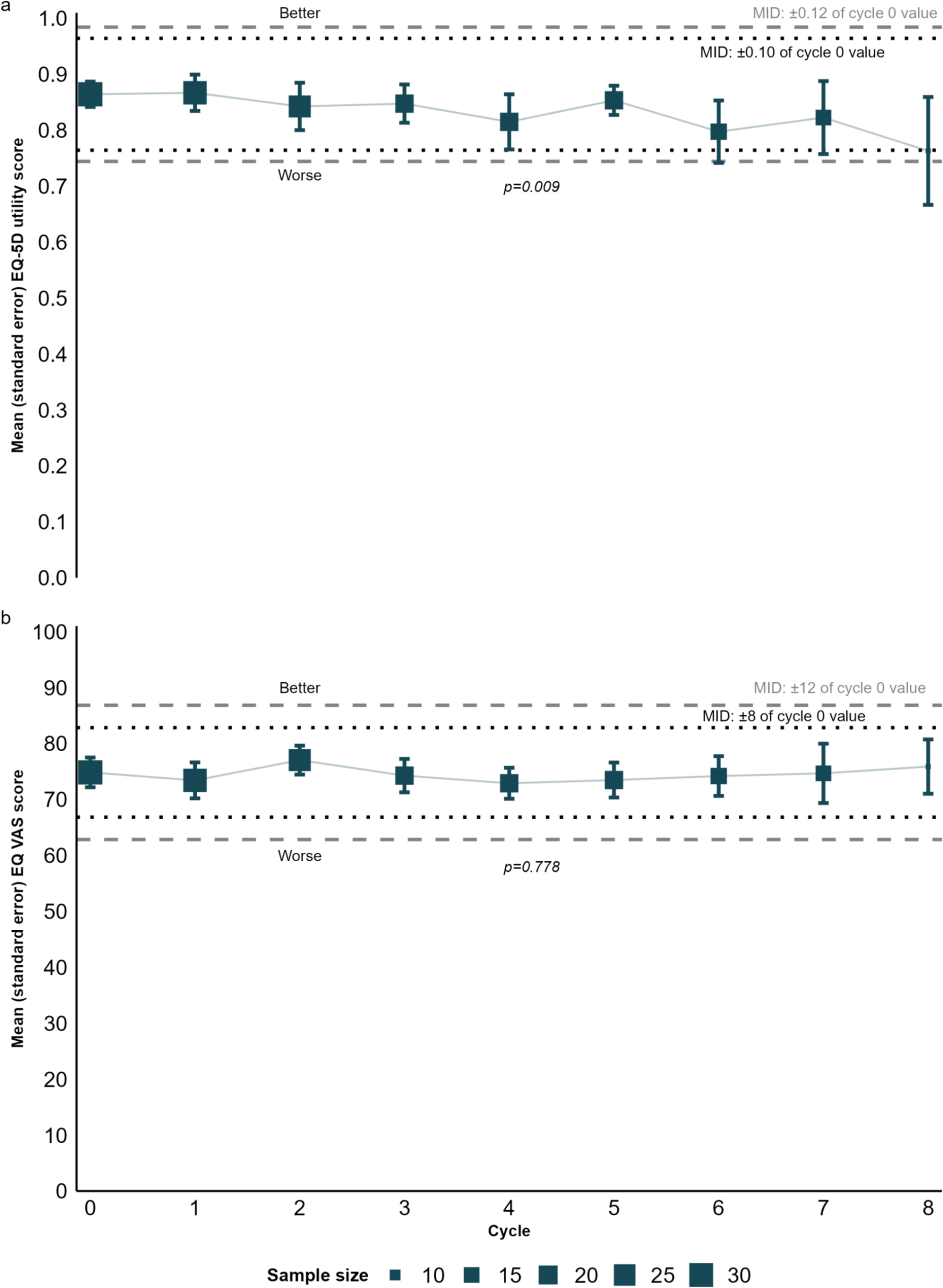
Changes in EQ-5D index value (**a**) and EQ VAS (**b**). MID, Minimally Important Difference; VAS, Visual Analog Scale. MIDs taken from Pickard et al. [24]. P-values refer to time (cycle) as an explanatory variable for scores

The EQ-5D-3L index value (Fig. 3a and Online Resource 1) and EQ VAS value (Fig. 3b and Online Resource 2) remained stable over time. The EQ-5D-3L index values decreased slightly over time, without exceeding the MID. Similarly, a slight drop, again not exceeding the MID, was observed in cycle 8. The mean change per cycle was − 0.011 (p-value = 0.009). Results did not change materially when adjusting for age and ECOG (see Online Resource 3). For the EQ VAS, no clinically meaningful decline was observed. The mean change per cycle was − 0.102 (p-value = 0.778). Including age and ECOG in the linear mixed model did not materially change results. Unadjusted models were generally preferred based on goodness-of-fit criteria, although differences in these criteria between unadjusted and adjusted models were generally small (see Online Resource 4).

#### FACT-General/FACT-Hep

**Fig. 4:**
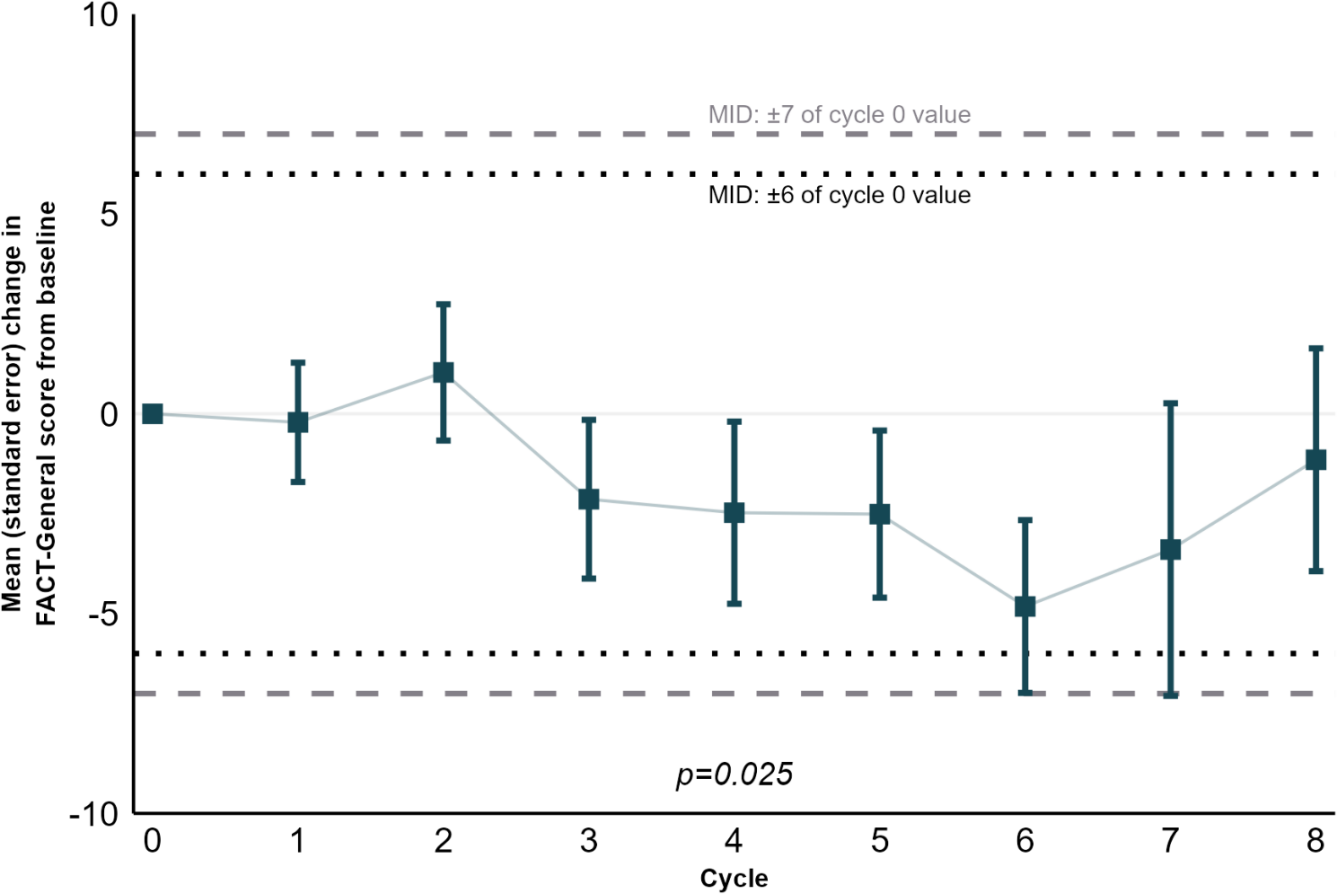
Change in FACT-General score. MID, Minimally Important Difference. MIDs taken from Steel et al. [23]. P-value refers to time (cycle) as an explanatory variable for the score

The FACT-General score remained stable between the baseline visit and cycle 8 although a statistically and clinically insignificant, transient deterioration in scores was observed between cycles 5 and 7 (Fig. 4).

**Fig. 5:**
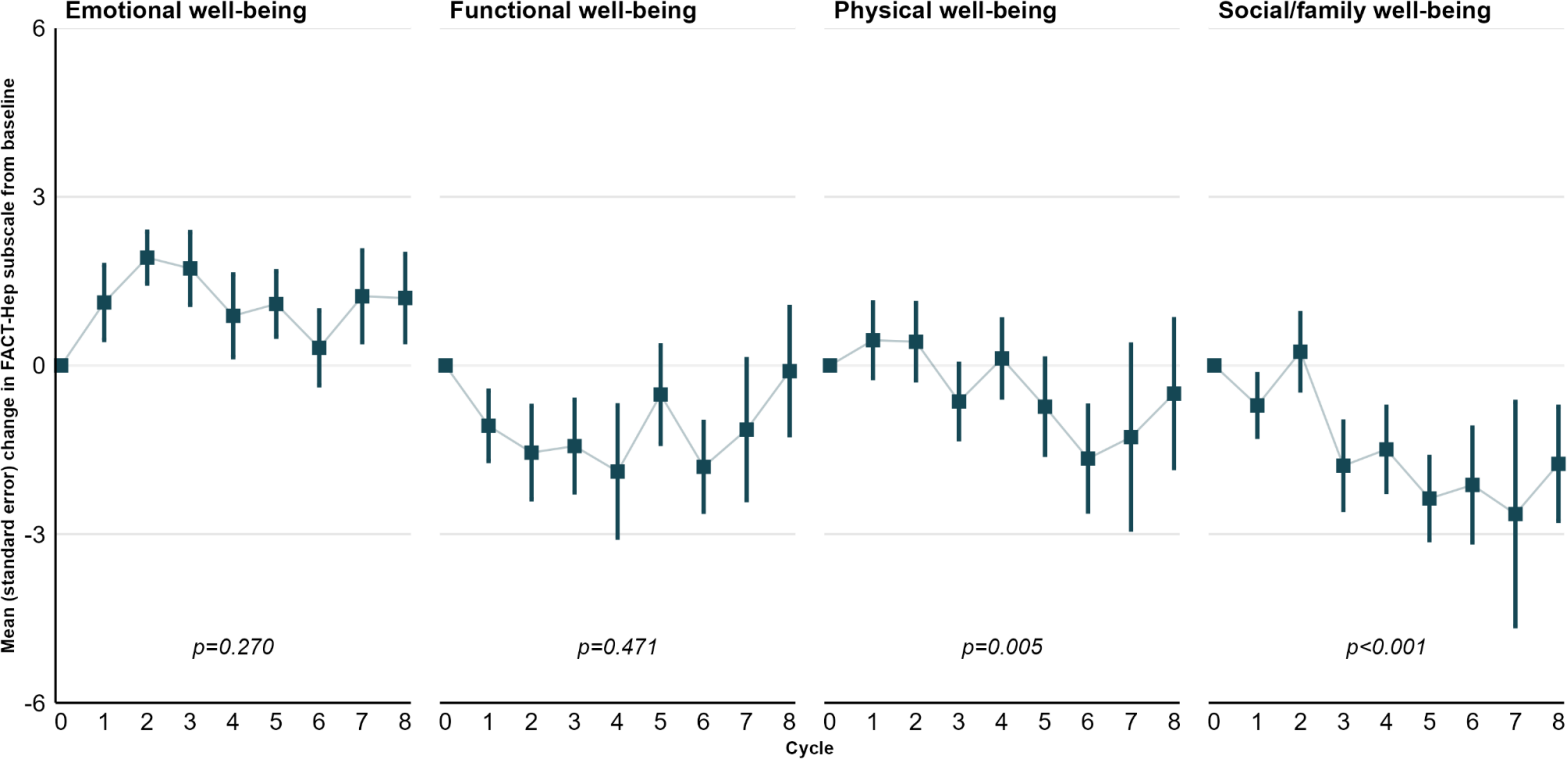
Changes in FACT-Hep subscale scores. P-value refers to time (cycle) as an explanatory variable for the score

The FACT-Hep subscales also remained broadly stable, despite a statistically and clinically significant but transient decline in SWB scores between cycles 5 and 7 (Fig. 5, p-value < 0.001).

Transient declines were also observed for PWB and FWB scores, but were neither statistically significant nor did they exceed clinical MIDs. The transient increase for EWB failed to reach clinical and statistical significance. In models adjusted for age and ECOG, neither predictor reached statistical significance (Online Resource 5).

#### TTD for FACT-Hep total score and HCS

**Fig. 6:**
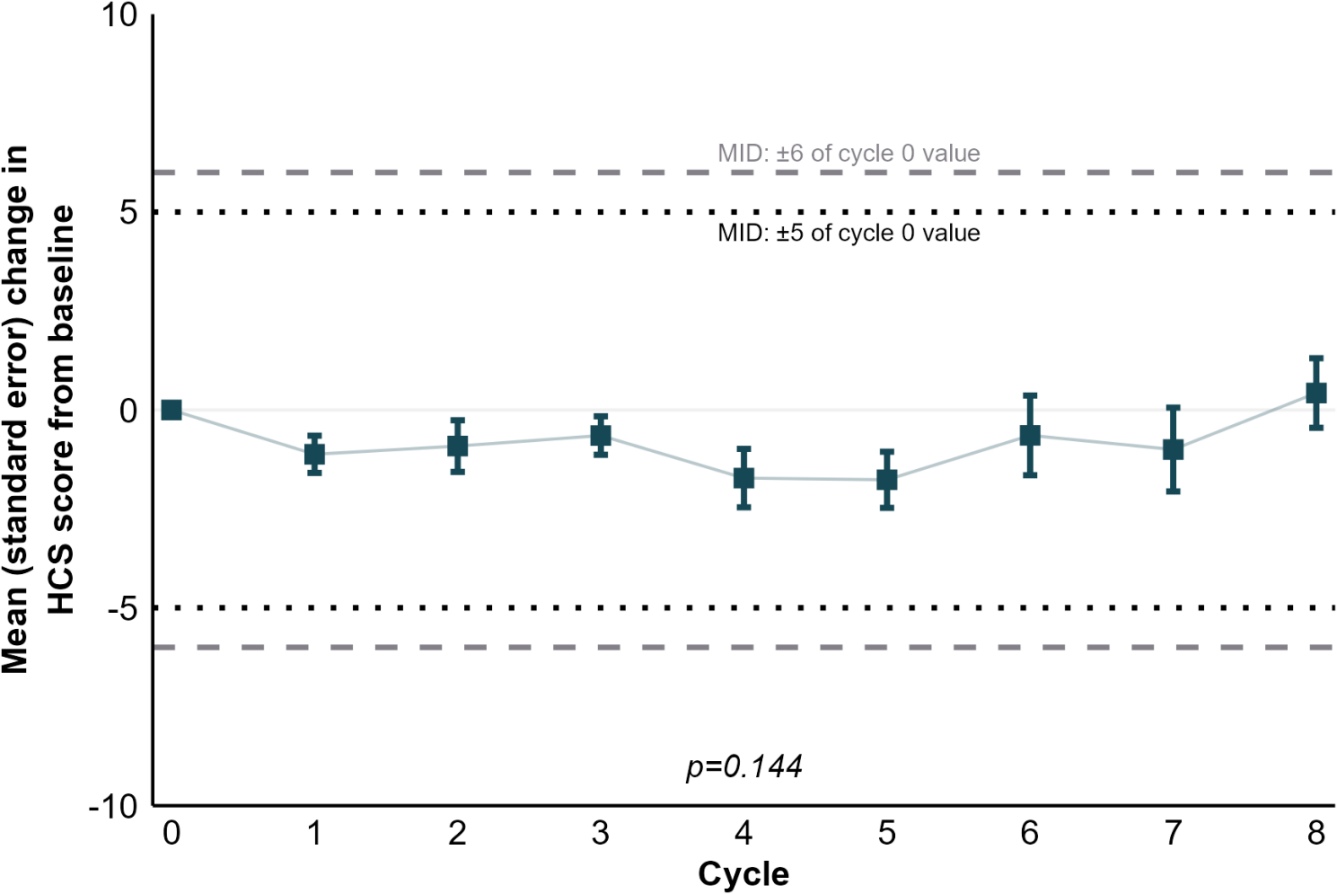
Changes in HCS scores. HCS, Hepatobiliary Cancer Scale; MID, Minimally Important Difference. MIDs taken from Steel et al. [23]. P-value refers to time (cycle) as an explanatory variable for the score

The FACT-Hep total score also remained largely unchanged between baseline and cycle 8 (Online Resource 6). Notably, while changes did not exceed MIDs, there was a statistically insignificant decline in scores between cycles 2 and 6 (*p* = 0.023). The median TTD in the FACT-Hep total score was 5.5 months (95% CI: 5.5 to *not reached*) (Online Resource 7). Similarly, HCS scores were stable, as the small fluctuations reached neither clinical nor statistical significance (Fig. 6). For HCS scores, the median TTD was not reached (Online Resource 8).

## Discussion

The present analysis is the first to report HRQoL data for the combination of SIRT with nivolumab, based on the NASIR-HCC trial [18]. Across the EQ-5D-3 L, EQ-VAS, and FACT-Hep instruments, HRQoL remained stable from baseline to the eight nivolumab cycle. Transient declines were observed between cycles 3 and 6 in patient-reported FACT-Hep, FACT-General, PWB, and SWB scores, but mean changes were not clinically meaningful at most evaluable time points. These results were aligned with the literature on the HRQoL impact of immunotherapies and SIRT.

Monotherapy with ICIs was associated with improved PROs and longer time to deterioration relative to control groups in a meta-analysis of nineteen randomized controlled trials across different types of cancer, suggesting a potential for combination with other systemic drugs [27]. For first-line treatment of unresectable HCC, clinically and statistically significant improvements over sorafenib were reported for nivolumab [13, 28] and for tislelizumab, including for fatigue and physical functioning [29]. Preserved HRQoL and similar TTDs were also seen in second-line treatment with pembrolizumab relative to best supportive care [30]. Furthermore, benefits were shown for combination immunotherapies. The combination of atezolizumab with bevacizumab reduced the risk of deterioration in generic cancer and disease-specific symptom scales relative to sorafenib [31] and these differences were deemed clinically meaningful. Similarly, first-line treatment with combined tremelimumab and durvalumab reduced the risk of decreased HRQoL relative to sorafenib [32].

Studies comparing SIRT with Y90 resin microspheres versus sorafenib have also reported good QoL profiles for SIRT, including the SIRveNIB trial, in which no statistically significant differences were observed between SIRT and sorafenib regarding EQ-5D [33]. In the SARAH trial, the EORTC QLQ-C30 global health status score was higher in patients receiving SIRT, and the benefit of SIRT was shown to increase over time [34, 35]. The observational CIRT study confirmed stable global health status scores following SIRT with Y90 resin microspheres over 24 months of follow-up [36]. In addition, a matching-adjusted indirect comparison [37] estimated similar TTD in quality of life for SIRT using Y90 resin microspheres, based on data from SARAH [34], and for atezolizumab-bevacizumab, based on data from the IMbrave150 study [9]. The analysis suggested numerically longer TTD for atezolizumab-bevacizumab, but the corresponding hazard ratio (HR 1.06 [95% CI 0.75 to 1.50], *p* = 0.725) did not meet conventional levels of statistical significance. In sensitivity analyses, SIRT was associated with longer TTD in QoL than atezolizumab-bevacizumab, although the difference was not statistically significant (HR 0.66 [95% CI 0.36 to 1.19], *p* = 0.163) [37].

These results on the HRQoL profiles of both SIRT and immunotherapy were confirmed by the present analysis of PROM data from the NASIR-HCC study, which adds to the growing literature on the HRQoL effects associated with locoregional and immunotherapeutic HCC treatments [38]. Data such as those provided here can inform patient decision-making, in which quality of life and the avoidance of treatment side effects have been shown to feature prominently, to the extent that patients are willing to trade survival for quality of life [39].

This is particularly relevant in the current scenario where multiple options are available in any first-line setting and upon tumor progression. The availability of PROM and HRQoL data for SIRT and immunotherapies, for resection (with only small differences between types of resection [40] and a long-term improvement or at least a return to baseline [41, 42]), for stereotactic body radiotherapy (without overall changes [43]), and for regorafenib (which delayed time to definitive deterioration relative to placebo [44]) reflect the growing importance of and interest in HRQoL in the field of HCC [20, 21]. This includes an increased understanding of the detrimental impact of HCC on HRQoL. Patients with HCC, even at earlier stages of the disease, generally have lower HRQoL than the general population as they commonly suffer from pain, fatigue/lack of energy, distress, reduced appetite, and sleeping problems [45, 46]. In addition, the disease negatively affects patients’ mental health as patients frequently experience fear, frustration, and depression [46–48]. Qualitative studies have demonstrated that physical symptoms and treatment side effects present a substantial burden on patients, who are often overwhelmed by their diagnosis and impacted in their spiritual well-being as they are forced to cope with changes in their body, self-perception, and sense of control [48, 49].

An important driver of reduced HRQoL in HCC is the loss of hepatic function. Recent US data suggested that Child-Pugh score (a measure of liver dysfunction) was associated with HRQoL measured using the FACT-Hep questionnaire [50]. Individuals in Child-Pugh class B reported more impairments in the FACT-Hep total score and across sub-scales than patients in class A. Reductions in HRQoL have also been reported for individuals with decompensated cirrhosis relative to non-cirrhotic individuals and those with compensated cirrhosis, independently of HCC, implying that the risk of HRQoL deterioration increases with decreasing liver function [51]. Sarcopenia, which occurs in up to 42% of HCC cases [52], also reduces HRQoL in patients with HCC post-resection, in addition to increasing the risk of clinical complications and of mortality following surgery [53, 54].

Quality of life, in turn, has been identified as a predictor of OS in HCC. In line with findings that PROMs prognosticate OS independently of clinical data in oncology in general [55], multiple studies of patients with HCC showed that better HRQoL and functioning were associated with prolonged OS [56, 57]. This pattern was observed in newly diagnosed HCC across stages [58], advanced HCC, including recurrent and palliative disease [56, 57, 59], and HCC with portal vein thrombosis [60].

The present analysis is not without limitations, as it was based on only thirty-two patients from a non-randomized study conducted in a single country. While the effect of treatment on HRQoL over time was explored in different models and robust across instruments and model specifications, readers should consider the limitations of the small sample size when using these data [61], e.g., in health economic analysis [62]. In addition, care should be taken not to extrapolate results beyond patients with unresectable HCC, as even small differences in study target populations can lead to potentially large effects on quality of life estimates [63]. As noted for NASIR-HCC safety and effectiveness outcomes, having all SIRT procedures performed at a single center ensured consistent treatment standards and therefore likely contributed to internal validity, but external validity and generalizability to other settings, including outside Spain, may be limited [18]. Given the observed pattern among some of the few patients reporting extreme problems at one visit not providing a subsequent response (Fig. 2), the possibility of a slight overestimation of HRQoL at later time points cannot be excluded, but this pattern would not have affected the overall conclusion of HRQoL stability over time.

## Conclusions

Following treatment of unresectable HCC with SIRT using Y90 resin microspheres and nivolumab, HRQoL, measured using the EQ-5D-3 L and FACT-Hep, remained stable over eight nivolumab cycles in the NASIR-HCC study. Combined with the promising clinical results from NASIR-HCC and similar studies, the good HRQoL profile suggests that combined SIRT and nivolumab should be investigated further as a treatment option in this difficult-to-treat patient population.

## Electronic supplementary material

Below is the link to the electronic supplementary material.

Supplementary Material 1

## Data Availability

All data relevant to the study are included in the article or uploaded as online supplemental information. No additional data can be made available as they are confidential patient records.

## Abbreviations

AIC: Akaike information criterion
BCLC: Barcelona clinic liver cancer
BIC: Bayesian information criterion
CI: Confidence interval
ECOG: Eastern cooperative oncology group
EORTC QLQ-C30: European organisation for research and treatment of cancer quality of life questionnaire C30
EQ-5D-3L/VAS: EuroQol 5 dimensions 3 levels/ visual analogue scale
EWB: Emotional wellbeing
FACT-G/Hep: Functional assessment of cancer therapy-general/ hepatobiliary
FWB: Functional wellbeing
HCC: Hepatocellular carcinoma
HCS: Hepatobiliary cancer subscale
HRQoL: Health-Related quality of life
ICIs: Immune checkpoint inhibitors
MID: Minimally important difference
OS: Overall survival
PROM: Patient-reported outcomes measure
PWB: Physical wellbeing
SIRT: Selective internal radiation therapy
SWB: Social and family wellbeing
TACE: Transarterial chemoembolization
TTD: Time to treatment deterioration
US: United States
Y90: Yttrium-90
