# Supplementary material for: Health-related quality of life in patients with unresectable hepatocellular carcinoma treated with SIRT and nivolumab: a sub-analysis of the NASIR-HCC trial
Source: J Patient Rep Outcomes. 2025 Apr 8;9:39. doi: 10.1186/s41687-025-00873-6 (PMC11978598; doi:10.1186/s41687-025-00873-6)

| **HEALTH-RELATED QUALITY OF LIFE IN PATIENTS WITH UNRESECTABLE HEPATOCELLULAR CARCINOMA TREATED WITH SIRT AND NIVOLUMAB: A SUB-ANALYSIS OF THE NASIR-HCC TRIAL**  **ELECTRONIC SUPPLEMENTARY MATERIAL** |
| --- |

**Abbreviations Used in this Electronic Supplementary Material**

| AIC | Akaike Information Criterion |
| --- | --- |
| ANOVA | Analysis of Variance |
| BIC | Bayesian Information Criterion |
| ECOG | Eastern Cooperative Oncology Group |
| TTD | Time to Discontinuation |

# Online Resource 1: EQ-5D-3L Index Values Over Time

| Cycle | Mean | Standard deviation | N |
| --- | --- | --- | --- |
| 0 | 0.864 | 0.126 | 31 |
| 1 | 0.867 | 0.175 | 29 |
| 2 | 0.843 | 0.216 | 26 |
| 3 | 0.848 | 0.149 | 19 |
| 4 | 0.815 | 0.215 | 19 |
| 5 | 0.854 | 0.114 | 19 |
| 6 | 0.798 | 0.223 | 16 |
| 7 | 0.823 | 0.253 | 15 |
| 8 | 0.763 | 0.273 | 8 |

# Online Resource 2: EQ VAS Over Time

| Cycle | Mean | Standard deviation | N |
| --- | --- | --- | --- |
| 0 | 74.8 | 14.8 | 31 |
| 1 | 73.4 | 17.3 | 29 |
| 2 | 77.0 | 13.2 | 26 |
| 3 | 74.3 | 13.0 | 19 |
| 4 | 72.9 | 12.1 | 19 |
| 5 | 73.5 | 13.6 | 19 |
| 6 | 74.2 | 14.2 | 16 |
| 7 | 74.7 | 20.5 | 15 |
| 8 | 75.9 | 13.7 | 8 |

# Online Resource 3: Mixed Linear Models for EQ-5D-3L Index Values

|  | Estimate | p-value |
| --- | --- | --- |
| *Unadjusted model* |  |  |
| Intercept | 0.871 | <0.001 |
| Time (cycle) | –0.011 | 0.009 |
| *Goodness of fit* | *AIC: –156.7; BIC: –143.9* | |
| *Adjusted model* |  |  |
| Intercept | 0.931 | <0.001 |
| Time (cycle) | –0.012 | 0.006 |
| Age | –0.215 | 0.037 |
| ECOG | –0.001 | 0.874 |
| *Goodness of fit* | *AIC: –157.6; BIC: –138.3* | |
| *ANOVA* | *p-value: 0.087* | |

# Online Resource 4: Mixed Linear Models for EQ Visual Analog Scale

|  | Estimate | p-value |
| --- | --- | --- |
| *Unadjusted model* |  |  |
| Intercept | 74.772 | <0.001 |
| Time (cycle) | –0.102 | 0.778 |
| *Goodness of fit* | *AIC: –1,448; BIC: –1,461.6* | |
| *Adjusted model* |  |  |
| Intercept | 74.414 | <0.001 |
| Time (cycle) | –0.117 | 0.749 |
| Age | –3.452 | 0.656 |
| ECOG | 0.010 | 0.972 |
| *Goodness of fit* | *AIC: –1,452.6; BIC: –1,471.8* | |
| *ANOVA* | *p-value: 0.893* | |

# Online Resource 5: Mixed Linear Models for FACT-General and Sub-Scales

| Model | Estimate | p-value |
| --- | --- | --- |
| **FACT-General total score** | | |
| *Unadjusted model* |  |  |
| Intercept | 0.250 | 0.841 |
| Time (cycle) | −0.484 | 0.025 |
| *Goodness of fit* | *AIC: –1,260.8; BIC: –1,273.7* | |
| *Adjusted model* |  |  |
| Intercept | 5.071 | 0.632 |
| Time (cycle) | −0.464 | 0.032 |
| Age | 5.419 | 0.181 |
| ECOG | −0.080 | 0.612 |
| *Goodness of fit* | *AIC: –1,262.7; BIC: –1,281.9* | |
| *ANOVA* | *p-value: 0.336* | |
| **FACT-Hep total score** | | |
| *Unadjusted model* |  |  |
| Intercept | 0.344 | 0.818 |
| Time (cycle) | −0.626 | 0.023 |
| *Goodness of fit* | *AIC = 1,094.3; BIC = 1,106.4* | |
| *Adjusted model* |  |  |
| Intercept | 13.416 | 0.249 |
| Time (cycle) | −0.623 | 0.024 |
| Age | 8.065 | 0.120 |
| ECOG | −0.207 | 0.237 |
| *Goodness of fit* | *AIC = 1,094.5 ; BIC = 1,112.6* | |
| *ANOVA* | *p-value: 0.148* | |
| **Physical Well-being subscale** | | |
| *Unadjusted model* |  |  |
| Intercept | 0.374 | 0.472 |
| Time (cycle) | −0.239 | 0.005 |
| *Goodness of fit* | *AIC: 965.70; BIC: 978.68* | |
| *Adjusted model* |  |  |
| Intercept | −5.571 | 0.165 |
| Time (cycle) | −0.211 | 0.013 |
| Age | 3.388 | 0.039 |
| ECOG | 0.086 | 0.158 |
| *Goodness of fit* | *AIC: 962.74; BIC:982.23* | |
| *ANOVA* | *p-value: 0.031* | |
| **Social/Family Well-being subscale** | | |
| *Unadjusted model* |  |  |
| Intercept | −0.057 | 0.911 |
| Time (cycle) | −0.308 | <0.001 |
| *Goodness of fit* | *AIC = 981.02; BIC = 994.01* | |
| *Adjusted model* |  |  |
| Intercept | 1.625 | 0.685 |
| Time (cycle) | −0.302 | <0.001 |
| Age | 1.841 | 0.269 |
| ECOG | −0.029 | 0.642 |
| *Goodness of fit* | *AIC = 983.50 ; BIC = 1,002.98* | |
| *ANOVA* | *p-value: 0.468* | |
| **Emotional Well-being subscale** | | |
| *Unadjusted model* |  |  |
| Intercept | 0.826 | 0.038 |
| Time (cycle) | 0.079 | 0.270 |
| *Goodness of fit* | *AIC = 863.77; BIC = 876.63* | |
| *Adjusted model* |  |  |
| Intercept | 6.064 | 0.064 |
| Time (cycle) | 0.071 | 0.327 |
| Age | −0.043 | 0.972 |
| ECOG | −0.079 | 0.106 |
| *Goodness of fit* | *AIC = 864.82 ; BIC = 884.11* | |
| *ANOVA* | *p-value: 0.229* | |
| **Functional Well-being subscale** | | |
| *Unadjusted model* |  |  |
| Intercept | −0.834 | 0.129 |
| Time (cycle) | −0.066 | 0.471 |
| *Goodness of fit* | *AIC = 958.79 ; BIC = 971.65* | |
| *Adjusted model* |  |  |
| Intercept | 3.109 | 0.510 |
| Time (cycle) | −0.070 | 0.451 |
| Age | 0.181 | 0.919 |
| ECOG | −0.060 | 0.400 |
| *Goodness of fit* | *AIC = 961.98 ; BIC = 981.27* | |
| *ANOVA* | *p-value: 0.668* | |
| **Hepatobiliary Cancer subscale** | | |
| *Unadjusted model* |  |  |
| Intercept | −0.602 | 0.175 |
| Time (cycle) | −0.111 | 0.144 |
| *Goodness of fit* | *AIC = 745.28; BIC = 757.59* | |
| *Adjusted model* |  |  |
| Intercept | 4.123 | 0.198 |
| Time (cycle) | −0.110 | 0.148 |
| Age | 3.022 | 0.048 |
| ECOG | −0.075 | 0.123 |
| *Goodness of fit* | *AIC = 743.17 ; BIC = 761.62.* | |
| *ANOVA* | *p-value: 0.047* | |

# Online Resource 6: Change in FACT-Hep Scores


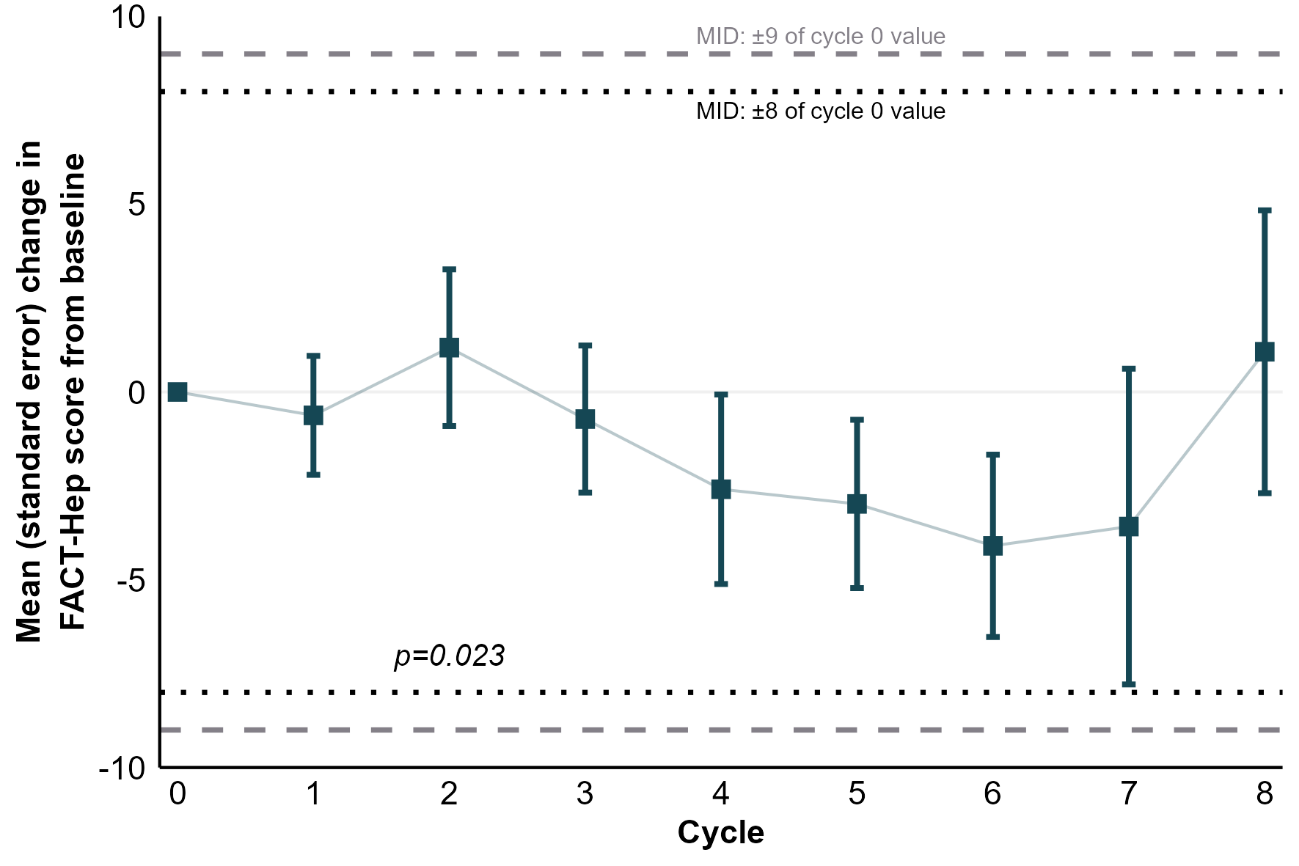


Abbreviation: MID, Minimally Important Difference. MIDs taken from Steel *et al.* [22]. P-value refers to time (cycle) as an explanatory variable for the score.

# Online Resource 7: Time to Deterioration in FACT-Hep Scores


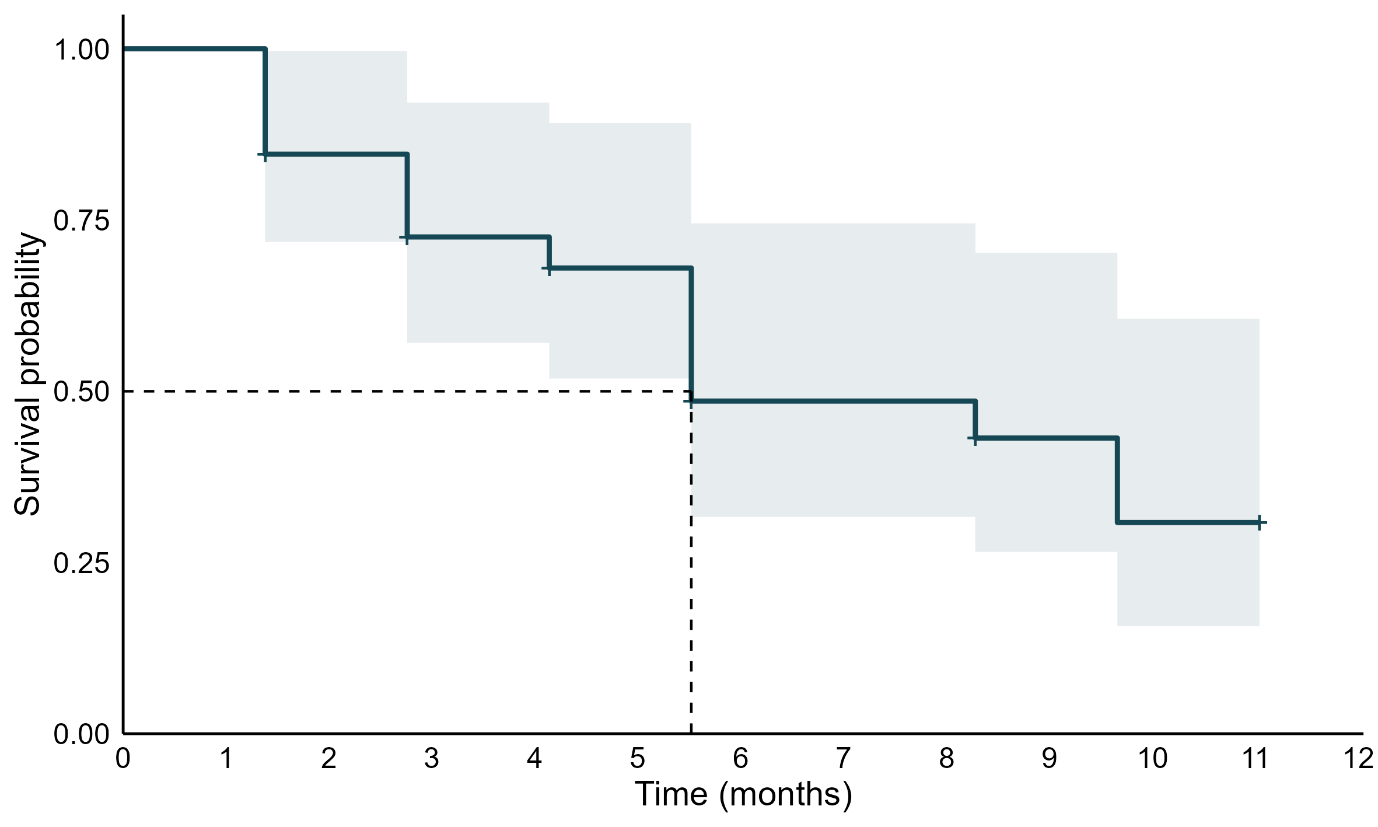


# Online Resource 8: Time to Deterioration in HCS Scores


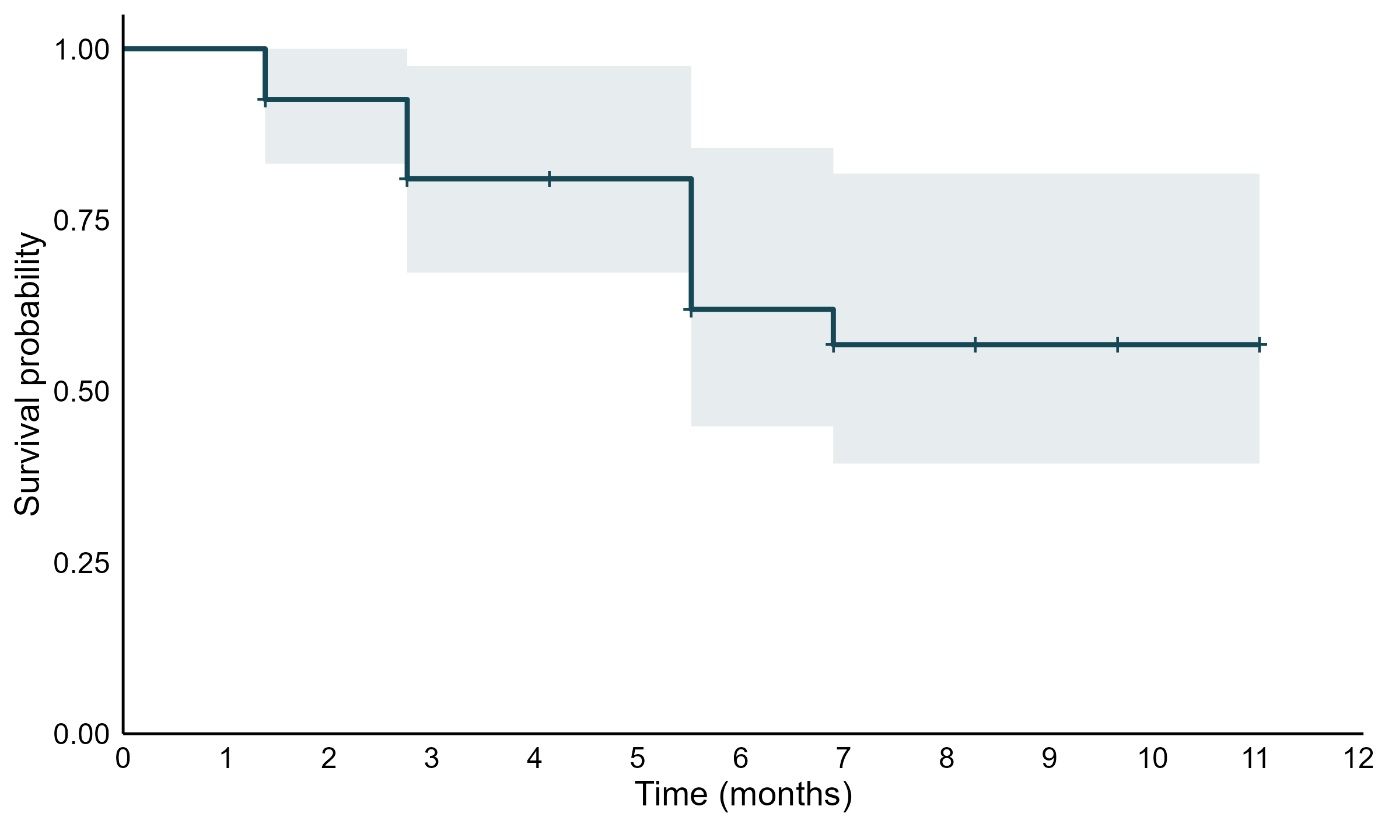

Supplement: Supplementary file 1 — Supplementary Material 1 [file 41687_2025_873_MOESM1_ESM.docx]
